# *Legionella pneumophila* and Protozoan Hosts: Implications for the Control of Hospital and Potable Water Systems

**DOI:** 10.3390/pathogens9040286

**Published:** 2020-04-15

**Authors:** Muhammad Atif Nisar, Kirstin E. Ross, Melissa H. Brown, Richard Bentham, Harriet Whiley

**Affiliations:** College of Science and Engineering, Flinders University, GPO Box 2100, Adelaide 5001, Australia; Muhammadatif.Nisar@flinders.edu.au (M.A.N.); Kirstin.Ross@flinders.edu.au (K.E.R.); Melissa.Brown@flinders.edu.au (M.H.B.); Richard.Bentham@flinders.edu.au (R.B.)

**Keywords:** *Legionella pneumophila*, protozoa, *Vermamoeba*, *Acanthamoeba*, potable water, hospital water, water disinfection, legionellosis

## Abstract

*Legionella pneumophila* is an opportunistic waterborne pathogen of public health concern. It is the causative agent of Legionnaires’ disease (LD) and Pontiac fever and is ubiquitous in manufactured water systems, where protozoan hosts and complex microbial communities provide protection from disinfection procedures. This review collates the literature describing interactions between *L. pneumophila* and protozoan hosts in hospital and municipal potable water distribution systems. The effectiveness of currently available water disinfection protocols to control *L. pneumophila* and its protozoan hosts is explored. The studies identified in this systematic literature review demonstrated the failure of common disinfection procedures to achieve long term elimination of *L. pneumophila* and protozoan hosts from potable water. It has been demonstrated that protozoan hosts facilitate the intracellular replication and packaging of viable *L. pneumophila* in infectious vesicles; whereas, cyst-forming protozoans provide protection from prolonged environmental stress. Disinfection procedures and protozoan hosts also facilitate biogenesis of viable but non-culturable (VBNC) *L. pneumophila* which have been shown to be highly resistant to many water disinfection protocols. In conclusion, a better understanding of *L. pneumophila*-protozoan interactions and the structure of complex microbial biofilms is required for the improved management of *L. pneumophila* and the prevention of LD.

## 1. Introduction

*Legionella pneumophila* is an opportunistic pathogen associated with community-acquired and nosocomial infections. It is the causative agent of legionellosis, which includes Legionnaires’ disease (LD), a severe atypical pneumonia infection, and Pontiac fever, an acute “flu-like” illness [1]. Globally, the incidence of LD has been increasing. In Europe, the number of notified cases increased from 4921 in 2011 to 11,343 in 2018 [2]. In the US, the number of notified LD cases has increased from 2301 in 2005 [3] to 7104 in 2018 [4], a 300% increase. Globally, the fatality rate of LD ranges from 2.2–10.3%, with the lowest in Singapore and the highest in European countries [5]. In nosocomial outbreaks the fatality rate can reach up to 48% [6,7,8].

The genus *Legionella* is comprised of 60 species and 80 distinct serogroups [9]. Globally, *L. pneumophila* is the primary aetiological agent of LD. In Europe and the US, *L. pneumophila* serogroup (SG1) is responsible for 70–92% reported cases [8]. According to WHO, 20–30% infections are caused by other *L. pneumophila* serogroups and only 5–10% are caused by other *Legionella* species (*L. micdadei*, *L. bozemanii*, *L. dumoffii* and *L. longbeachae*) [10]. However, unlike rest of the world, in Australia and New Zealand, *L. longbeachae* is associated with ≈ 50% reported cases of legionellosis [11,12].

*L. pneumophila* is ubiquitous in manufactured water systems [10] and in the USA has been identified as the primary cause of all potable water related outbreaks [13]. Manufactured water systems, building plumbing systems, recreational water, cooling towers and humidifiers are major sources of *L. pneumophila* [10]. Inside these plumbing structures, *Legionella* and protozoan hosts are incorporated within biofilms. Factors like water stagnation, higher levels of organic carbon and moderate temperatures can increase the rate of biofilm formation [14,15]. Transmission occurs through inhalation or aspiration of contaminated aerosols or water [16]. *L. pneumophila* maintains long term contamination of manufactured water systems through its growth within protozoan hosts, association with biofilms and disinfectant resistance or tolerance [17,18]. Freshwater amoebae are the natural eukaryotic hosts of *Legionella*; whereas, humans are considered accidental hosts [19]. In the human body, *Legionella*–contaminated aerosols are inhaled into the lungs and phagocytosed by alveolar macrophages. The alveolar macrophages behave like amoebae hosts and facilitate the intracellular division and propagation of *Legionella*, resulting in LD [20,21].

Understanding the interactions between *L. pneumophila* and protozoan hosts is essential to inform water treatment and risk management strategies for the prevention of LD. Protozoan hosts play an important role in the ability of *L. pneumophila* to survive exposure to physiochemical and environmental stresses. Protozoans facilitate the intracellular replication and packaging of live bacterial cells in the stress resistant membrane bound infectious export vesicles [22,23]. The cysts of cyst-forming amoebae provide a protective shelter from prolonged environmental stress [24]. There are numerous reports describing existence of *L. pneumophila* harboring within protozoans from thermally-, chemically-, and UV radiation-treated potable water supplies and storage reservoirs [25]. Protozoan hosts and environmental stress may facilitate the genesis of highly resistant and potentially infectious viable but non-culturable (VBNC) *L. pneumophila* [26,27]. Importantly, water storage facilities and distribution networks of many countries have been shown to be highly contaminated with protozoans that may act as hosts for *L. pneumophila* (>0–4500 cell/L cell density) [28].

This systematic literature review collated studies which detected *L. pneumophila* in association/connection with protozoan hosts from hospital or municipal potable water distribution systems and discusses this relationship under diverse environmental conditions. The effectiveness of different physical and chemical water treatment methods to control the *L. pneumophila* and its protozoan hosts is described and implications for the control and management of these water distribution systems is explored.

## 2. Results

**Figure 1:**
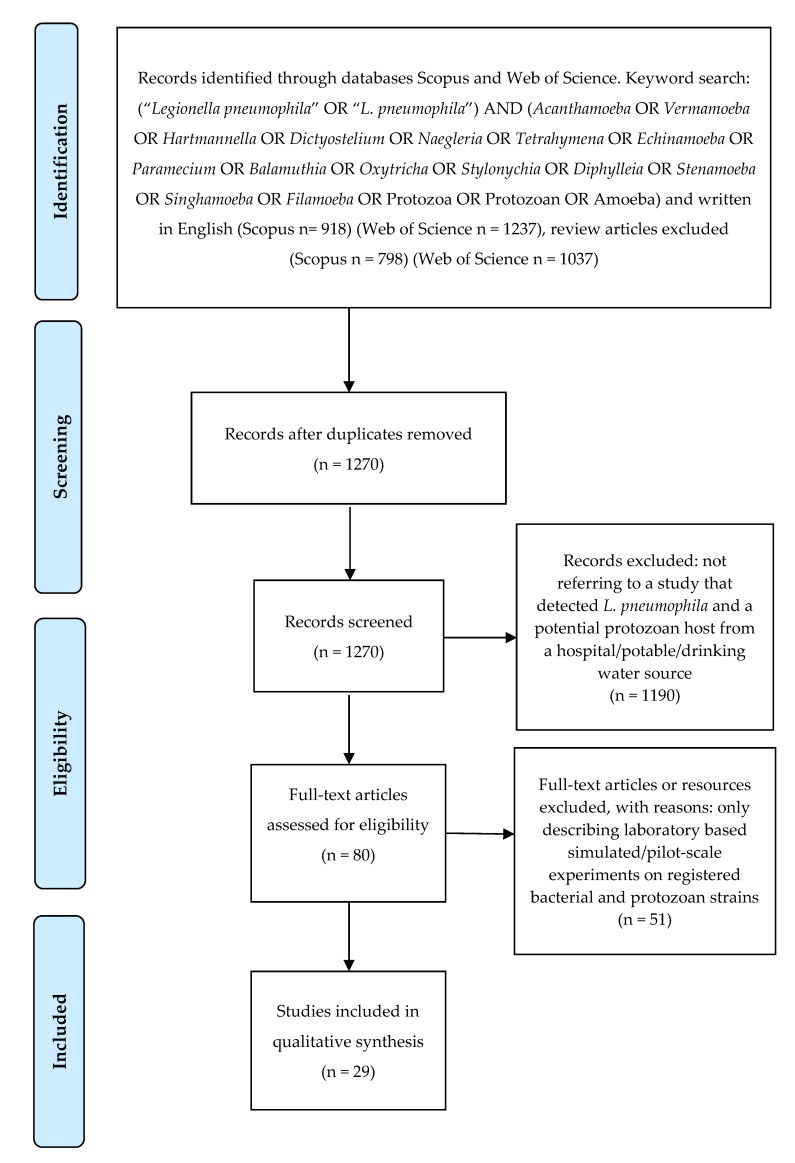
Overview of search methods and articles inclusion and exclusion criteria.

One thousand two hundred and seventy abstracts were obtained from the Web of Science and SCOPUS. After applying the described criteria (see Figure 1 and the Materials and Methods section), 29 research manuscripts discussing *L. pneumophila* and its protozoan hosts in hospital and potable water systems were included in the study (Table 1). Potential protozoan hosts playing crucial role(s) in the *L. pneumophila* life-cycle and living in both types of water systems are compiled in Table 2. These protozoan hosts have the potential to provide an appropriate habitat for replication and survival of *L. pneumophila*.

The articles from hospital settings showed that *L. pneumophila* Serogroup 1 (hereafter SG1) is the most common serogroup causing infection in USA and European countries. Globally, SG1 is also associated with community acquired legionellosis [29,30]. However, a limitation was that most municipal potable water supply studies did not characterize the *L. pneumophila* serogroups. To investigate the different *L. pneumophila*-protozoan interactions, some studies used co-isolation and co-culturing techniques or PCR. Other approaches included techniques like scanning electron microscopy or DVC-FISH to demonstrate the fate of internalized bacteria. The electron microscope studies conducted in hospital settings found that *L. pneumophila* SG1 is able to multiply inside *Echinamoeba exudans* [31] and *Vermamoeba vermiformis* (formerly *Hartmannella vermiformis*) [32]. Likewise, PCR-based examination of potable water also demonstrated the presence of *L. pneumophila* inside *V. vermiformis* [33]. Another study used DVC-FISH to detect intracellular *L. pneumophila* inside *Acanthamoeba* and *V. vermiformis* from a potable water supply [34]. Other studies (mentioned in the Table 1), demonstrated the co-existence of free-living *L. pneumophila* and protozoan hosts, but did not characterize the specific interaction or fate of internalized bacteria. The systematic literature review identified a more diverse number of potential protozoan hosts from hospitals compared with municipal potable water systems. This could be due to the more diverse dynamics of hospital water distributions systems (Table 2). The hosts identified in the hospital settings consisted of three phyla, five classes and twelve genera, whereas the hosts isolated from potable water consisted of only two phyla, three classes and five genera. Two genera of Amoebozoa namely, *Vermamoeba* and *Acanthamoeba*, are frequently reported from both types of facilities as potential hosts. Available literature demonstrated that non-cyst-forming and ciliated protozoans can also be potential hosts for *L. pneumophila*. Most of the studies were designed specifically to explore the interactions between *L. pneumophila* - *Vermamoeba*/*Acanthamoeba*, and the diversity and the role of other possible protozoans were not investigated.

In the studies identified, diverse physical and chemicals methods were used to disinfect the hospital and municipal potable water systems. Chlorination (<0.05–<4 mg/L) using different chlorine compounds was frequently reported as being used in both settings. Protozoans and *L. pneumophila* could still be isolated from both hospital and municipal potable water systems despite chlorination (<0.05–<4 mg/L), and/or ozonisation and thermal (<50–70 °C) disinfection protocols being in place. Importantly, several studies from hospital settings reported regular outbreaks of legionellosis. This represents a failure of existing disinfection protocols. The systematic literature review revealed that *L. pneumophila–Acanthamoeba*/*Vermamoeba* were extensively co-isolated from chlorinated and thermally treated water. This demonstrates the potential tolerance of *L. pneumophila* and protozoan hosts to survive under a wide range of disinfection conditions.

## 3. Discussion

The studies identified in this review have demonstrated the failure of many common disinfection protocols to achieve long term elimination of *L. pneumophila* from hospital and potable water supplies when protozoan hosts are present [35,38] (as mentioned in Table 1). This long term survival could be attributed to association with biofilms, inherent tolerance of *L. pneumophila* to high temperature and chemical disinfectants, and constant reseeding from source water [59]. However, perhaps the most interesting and undervalued relationship is the interactions with protozoan hosts. The studies identified (Table 1) are from 14 different countries, which demonstrates the need for further research to understand the *L. pneumophila*–protozoan interaction under different environmental conditions found globally. Proper management of legionellosis requires a better understanding of *L. pneumophila–*protozoan interaction, the diversity of protozoan hosts in hospital and potable water systems and the role of the host in bacterial survival under different environmental conditions.

### 3.1. Implications for the Control of L. pneumophila

Numerous studies have demonstrated the presence of *L. pneumophila* in disinfected water supplies [60,61]. An important factor enabling *L. pneumophila* survival in the built environment is its interaction with a protozoan host [62,63,64] (as mentioned in Table 3). Thermal treatment is one of the most common methods used to disinfect hospitals and building water supplies. In the USA [35], Germany [38] and Slovakia [41], thermal disinfection was adopted for management of nosocomial outbreaks of legionellosis. This strategy was unable to maintain water control for a long period of time [35,38] (as mentioned in Table 1). Rhoads et al. [64] reported that *L. pneumophila* associated with *V. vermiformis* can tolerate thermal (58 °C) treatment, and this disinfection protocol is unable to reduce microbial load in water. Published evidence suggests *Legionella* associated with *Acanthamoeba* are more thermos-tolerant and can survive at even higher temperatures ranging from 68–93 °C [63]. According to Steinert et al. [38] members of *L. pneumophila* SG1 are more thermo-tolerant than SG2. This is significant given the high number of legionellosis cases associated with *L. pneumophila* SG1.

As per WHO guidelines [65], 0.2 mg/L of free residual chlorine at point of delivery is recommended in potable water for disinfection. A pilot scale study conducted by Muraca et al. [66] demonstrated that 4 to 6 mg/L chlorine treatment for 6 h resulted in 5–6 log reduction of *L. pneumophila*. It was also observed that the efficacy of chlorine against *Legionella* was enhanced at 43 °C. However, at high temperatures a continuous flow of chlorine was required to overcome thermal decomposition. In vitro studies demonstrated higher level of tolerance to free chlorine (up to >50 mg/L) when bacteria are associated with host *Acanthamoeba* cysts [67]. According to Kool et al. [68], water disinfection with monochloramine resulted in a reduction of nosocomial LD outbreaks in USA. However, other studies have shown that some strains of *L. pneumophila* can tolerate high levels of monochloramine disinfection (17 mg-min/L for 3 log reduction) [69]. Donlan et al. [70] reported that *L. pneumophila* associated with amoebae in biofilm are less susceptible to chlorine and monochloramine treatment. It is also reported that monochloramine disinfection in hospital settings results in transformation of *L. pneumophila* vegetative cells to VBNC state [27].

According to Walker et al. [71] chlorine dioxide can effectively control *L. pneumophila* from hospital water system. In vitro studies demonstrated that 0.4 mg-min/L residual chlorine dioxide achieved a 3 log reduction of *L. pneumophila*. However, this procedure was not effective for amoebae associated *L. pneumophila* [69]. According to Schwartz et al. [72] *Legionella* biofilms on polyvinyl chloride, polyethylene and stainless steel materials can tolerate chlorine dioxide treatment. Muraca et al. [66] conducted a pilot scale study and reported that 1–2 mg/L residual concentration O_3_ treatment for 5 h resulted in 5 log reduction of *L. pneumophila*. However, half-life of ozone in water is very short, so it is difficult to maintain residual concentration in water supplies. According to Wang et al. [54], if chlorination and ozonisation is used in combination, it can target both *L. pneumophila* and its host protozoans effectively. In combination both treatments effectively eliminated planktonic *L. pneumophila* and free living *Naegleria* from water, whereas this combination could only reduce the population of *Acanthamoeba* (≈0.9 log_10_ gene copies/g). In comparison to chlorination alone, this combination method significantly reduced the population of *L. pneumophila* (≈3 log_10_ gene copies/g) and host amoebae (≈3 log_10_ *Naegleria* gene copies/g and ≈6.1 log_10_ *Acanthamoeba* gene copies/g) co-existing in biofilms.

UV irradiation is another method of disinfection. These radiations harbor strong genotoxic attributes. Cervero-Arago et al. [73] demonstrated that 5–6 mJ/cm^2^ UV dose was sufficient to achieve 4 log reduction *L. pneumophila* population. According to Muraca et al. [66] 30 mJ/cm^2^ UV rays treatment for 20 min resulted in 5 log reduction of *L. pneumophila*. However, continued exposure to same fluence rate for 6 h unable to eliminate all culturable *L. pneumophila* (1–2 × 10^2^ CFU/mL). Schwartz et al. [72] reported that *Legionella* biofilms on stainless steel, polyvinyl chloride and polyethylene surfaces can tolerate UV treatment. It was also reported that amoebae associated *L. pneumophila* can tolerate much higher doses of UV rays [73].

### 3.2. Protozoan Host Control Strategies

Protozoans present in water supplies play an important role in *L. pneumophila* survival and resistance against disinfection protocols. Interesting, it has also been suggested that some protozoans infected by *L. pneumophila* have increased resistance to disinfection procedures compared to those uninfected [74]. As such, an understanding of protozoan disinfectant resistance and *L.pneuophila–*protozoan interactions is essential for the improved management of manufactured water systems. According to Loret et al. [75], common water chemical disinfection protocols, i.e., ozonisation (0.5 mg/L), chlorination (free chlorine 2 mg/L), electro-chlorination (free chlorine 2 mg/L), monochloramine (free chlorine 2 mg/L), chlorine dioxide (0.5 mg/L) and Cu^+^/Ag^+^ ions (0.5/0.001 mg/L) treatments, are unable to completely eliminate amoebae cysts hosting *Legionella* from water supplies (Table 3). These methods appear to be only effective against the free living amoebae population, as they are not feasible for targeting biofilm-associated amoebae [76]. The non-standardized approach to evaluating disinfection limits is one of the gaps in knowledge raised in the discussion section.

In vitro studies have shown 1 mg/L chlorine is sufficient to inhibit the growth of *Acanthamoeba*, *Vermamoeba* and *Vahlkampfia* trophozoites. Importantly, after two hours exposure, chlorine produced complete die-off of trophozoites [77]. According to Kuchta et al. [78] 2–4 mg/L chlorine treatment for 30 min can completely inactivate *Vermamoeba* trophozoites. Whereas, trophozoites of some strains of *Hartmannella* required 15 mg-min/L chlorine treatment for only 2 log reduction [79]. Mogoa et al. [80] reported that *Acanthamoeba* trophozoites exposed to 5 mg/L chlorine for 30 s resulted in a 3 log population reduction. It was also demonstrated that in *Acanthamoeba*, chlorination induces various cellular changes including reduction in cell size and alterations in cellular permeability. Dupuy et al. [79] noticed that *Acanthamoeba* trophozoites treated with 28 mg/L chlorine for 1 min only resulted in a 2 log reduction. In comparison with uninfected *Acanthamoeba* trophozoites, *L. pneumophila* infected *Acanthamoeba* trophozoites were more resistant against sodium hypochlorite (1024 mg/L) treatment [74].

Generally, inactivation of *Acanthamoeba* and *Vermamoeba* cysts required 5 mg/L chlorine, whereas for *Vahlkampfia* 2 mg/L chlorine treatment. It is important to note that cysts of *Acanthamoeba* were found highly resistant and only a 2 log reduction was noticed after eight hours exposure [77]. It was also reported that *Acanthamoeba* cysts can tolerate 100 mg/L of chlorine for 10 min [81]. According to Dupuy et al. [79] treatment of *Acanthamoeba* cysts with 856 mg-min/L results in only 2 log reduction. Loret et al. [82] reported that to achieve 4 log reduction for *Acanthamoeba polyphaga* cysts 3500 mg-min/L chlorine treatment is required. Likewise certain strains of *Hartmannella* cysts can tolerate high dose of chlorine (2 log reduction by 156 mg-min/L) [79]. Exposure of *Vermamoeba* cysts to 15 mg/L of chlorine for 10 min was lethal and resulted in complete inactivation [83].

Unlike *Acanthamoeba* and *Vermamoeba*, trophozoites and cysts of *Naegleria* were found sensitive to available disinfection protocols. *Naegleria* trophozoites were sensitive to 0.79 mg/L chlorine treatment for 30 min [84], whereas cysts were inactivated after exposure to 1.5 mg/L chlorine for 1 h [85]. Dupuy et al. [79] reported that chlorine treatment of *Naegleria* trophozoites with 5 mg-min/L resulted in only 2 log reduction and cysts can tolerate much higher levels of chlorine (29 mg-min/L for 2 log reduction). In potable water *Naegleria fowleri* associated with biofilms was able to tolerate 20 mg/L chlorine for 3 h [86].

In comparison to chlorine, chloramine is regarded as more stable disinfectant and capable to penetrate complex biofilms [68]. Dupuy et al. [79] suggested that instead of chlorine, monochloramine is effective chemical disinfectants against trophozoites and cysts of *Acanthamoeba*, *Vermamoeba* and *Naegleria*. It is possible that monochloramine harbors greater penetrating power than chlorine and easily enter in trophozoites and cysts. According to Mogoa et al. [87] monochloramine specifically targets the cell surface of *Acanthamoeba*. Dupuy et al. [79] identified that 352 mg-min/L monochloramine exposure resulted in 2 log reduction of *Acanthamoeba* cysts. Goudot et al. [88] noticed that 4–17 mg/L monochloramine exposure for 1 min only resulted in 2 log reduction of both planktonic and biofilm associated *Naegleria*. According to Dupuy et al. [79] to achieve 2 log reduction of *Hartmannella* trophozoites and cysts 12 mg-min/L and 34 mg-min/L monochloramine dose is required, respectively. Although in vitro studies demonstrate that higher concentration of chlorine-based disinfectants can inhibit the proliferation of protozoans; however, it can corrode the plumbing system pipes.

Chlorine dioxide has been shown to easily penetrate into amoeba trophozoites and cysts and specifically promotes cytoplasmic vacuolization in *Acanthamoeba* [87]. However the efficacy of chlorine dioxide varies from amoeba strains. The cyst form of some *Acanthamoeba* strains have been demonstrated to be highly tolerant to chlorine dioxide (35 mg-min/L for 2 log reduction) [79]. Loret et al. [82] demonstrated that an 80 mg-min/L dose of chlorine dioxide is required to achieve 4 log reduction of *Acanthamoeba polyphaga* cysts. Importantly, most studies were designed to investigate the effect of disinfection procedures on amoeba and there are limited studies on *L. pneumophila*-amoebae interactions during disinfection.

Ozonisation is an effective method of water disinfection. According to Cursons et al. [84], a dose of ozone 6.75 mg/L (0.08 mg/L residual level after 30 min) was sufficient to kill 99.9% (3 log reduction) trophozoites of *Acanthamoeba* and *Naegleria*. However, biofilm associated *Acanthamoeba*, *Hartmannella*, and *Vahlkampfia* were always found resistant to such treatments [76]. Loret et al. [82] demonstrated that 10 mg-min/L ozone dose resulted in 3 log reduction of *Acanthamoeba* trophozoites, however cysts retained viability.

Thermal treatment is a common physical disinfection protocol used for potable water supplies. According to Chang [89] trophozoites of *Naegleria* can survive at 55 °C for 15 min, whereas cysts can tolerate 65 °C for 3 min. *Vermamoeba* trophozoites and cysts have been shown to be completely inactivated by exposure to 60 °C for 30 min [78,83]. Thermal treatment of *Acanthamoeba* trophozoites and cysts at 65 °C for 10 min resulted in full inactivation [90]. Loret et al. [82] demonstrated that thermal treatment of *Acanthamoeba polyphaga* cysts at 65 °C for 120 min resulted in 5 log reduction. However, Storey et al. [81] reported that *Acanthamoeba castellanii* cysts are thermally stable and retain viability at 80 °C for 10 min. It has also been reported that thermal treatment can enhance the efficiency of chlorination. Although at high temperature (50 °C) the solubility of chlorine gas in water decreases significantly and very corrosive to pipe work, but its amoebicidal activity increases slightly [69].

UV treatment is another method of disinfection recommended by WHO. As per recommendation in 10 mJ/cm^2^ dose is sufficient for 99.9% (3 log) inactivation of protozoans like *Giardia* and *Cryptosporidium* [65]. According to Cervero-Arago et al. [73] to achieve 3 log reduction of *V. vermiformis* trophozoites 26 mJ/cm^2^ UV dose was required, whereas 76.2 mJ/cm^2^ for cysts. It was also noticed that exposure to 72.2 mJ/cm^2^ irradiance resulted in 3 log reduction of *Acanthamoeba* trophozoites [73]. Aksozek et al. [91] reported viability of *Acanthamoeba castellanii* cysts after exposure to high doses of UV rays (800 mJ/cm^2^). According to Sarkar and Gerba [92] to achieve 4 log reduction of *Naegleria fowleri* trophozoites and cysts 24 mJ/cm^2^ and 121 mJ/cm^2^ UV irradiance is required, respectively. A pilot scale study conducted by Langmark et al. [93] demonstrated inability of UV irradiation to reduce biofilm associated amoebae. In contrast with other protozoans, members of the *Acanthamoeba* genera are more resistant to both chemical and physical disinfection protocols.

As per water quality guidelines of WHO [65], 41 mg-min/L chlorine at 25 °C OR 1000 mg-min/L monochloramine at 15 °C OR 7.3 mg-min/L chlorine dioxide 25 °C OR 0.63 mg-min/L O_3_ at 15 °C OR 10 mJ/cm^2^ UV rays, treatments are required for inactivation of pathogenic protozoan (reference protozoa *Giardia*), as mentioned earlier in this section protozoans facilitating growth of *L. pneumophila* can thrive in these conditions (Table 3).

So far, studies have investigated the efficacy of water disinfection protocols against *Acanthamoeba*, *Hartmannella*, *Naegleria* and *Vermamoeba*. However, there are numerous other waterborne cyst-forming, non-cyst forming and ciliated protozoans which support the proliferation of *L. pneumophila*. Therefore, there is a need for more research and a standardized approach to evaluating disinfection protocol(s) that target both *L. pneumophila* and potential protozoan hosts. According to our literature survey, the effectiveness of available disinfection protocols depends upon the species, strain and cellular state of protozoans, as well as the type of disinfection technique and exposure time.

### 3.3. Detection Methods

The most commonly used methods to investigate potential *L. pneumophila* protozoan hosts are co-culture and co-isolation assays [19]. The co-culture assay is widely used in the laboratory to study *Legionella*-protozoan interactions. In this method, *Legionella* is allowed to grow in a protozoan host and fate of bacterium is determined microscopically [94]. In vitro laboratory studies showed that *Acanthamoeba* [95] and *Tetrahymena* [96] allow intracellular replication and packaging of live *L. pneumophila* into export vesicles. Other protozoan genera; *Balamuthia* [97], *Dictyostelium* [98], *Echinamoeba* [31], *Naegleria* [99], *Paramecium* [100], and *Vermamoeba* [32], facilitate intracellular replication of *L. pneumophila*. The second method is used to detect naturally co-existing *Legionella*-protozoans from environment, but microscopically it is very difficult to find protozoans containing *Legionella* in the natural environment [101]. As an alternative approach, a sample is screened for the presence of both *Legionella* and protozoan hosts. Generally, samples are screened by PCR [102,103], fluorescence in situ hybridization [104], classical culturing techniques and microscopy [105,106]. These methods are good for screening environmental samples but are unable to delineate the underlying interactions between *Legionella* and host protozoans. Nowadays, PCR based protocols are widely used to detect *L. pneumophila* and protozoan hosts from engineered water systems. In comparison to classical culturing methods, these protocols are rapid and highly sensitive. However, most of the nucleic acid-based protocols are unable to differentiate viable and dead organisms. Propidium monoazide-PCR or ethidium monoazide-PCR are modified nucleic acid detection protocols to enumerate the live bacteria [107,108] and protozoan hosts [109,110]. To estimate burden of *L. pneumophila* and protozoan hosts in water distribution system, it is necessary to measure the quantity of alive and dead organisms regularly. This literature review demonstrates that *Vermamoeba* and *Acanthamoeba* are predominant hosts of *L. pneumophila* in the context of hospital and potable water systems. Many cyst-forming, non-cyst forming and ciliated protozoans have been found associated with *L. pneumophila* and are identified as potential hosts; however, in vitro co-culture assays and microscopic studies are required for confirmation and characterization of this interaction.

During stress (i.e., thermal, nutrient, chemical and radiation), *L. pneumophila* can enter into a VBNC state. After the end of such a stress period, in presence of a suitable host or favorable environmental conditions, the VBNC state can transform back into metabolically active cellular state [111]. Importantly, the underlying mechanisms of resuscitation from VBNC are not yet well understood. However, as the VBNC form is by definition a non-culturable state, classical microbiology culturing techniques cannot be used to monitor viability. Thus, in vitro co-culture assays can be used to resuscitate VBNC in the laboratory [74]. Alternative approaches to analyze VBNC are the analysis of membrane integrity and molecular screening [112]. There are also studies that have shown that intracellular replication of *L. pneumophila* induces VBNC state. According to Buse et al. [26] transformation of *V. vermiformis* trophozoites into cysts promotes biogenesis of VBNC *L. pneumophila*. Therefore, the interaction with protozoan hosts may also affect the ability to monitor the efficacy of disinfection protocols against *L. pneumophila*, because the bacteria may be in the VBNC form. Available literature only discusses disinfection protocols, which target culturable *L. pneumophila*. To our knowledge, there are limited studies investigating the effectiveness of disinfection protocols to eliminate VBNC *L. pneumophila*. It is our suggestion to adopt membrane integrity and in vitro co-culture assays to evaluate the disinfection procedure against VBNC *L. pneumophila*.

## 4. Materials and Methods

The databases Scopus and Web of Science were searched for articles written in English containing the keywords (“*Legionella pneumophila*” OR “*L. pneumophila*”) AND (*Acanthamoeba* OR *Vermamoeba* OR *Hartmannella* OR *Dictyostelium* OR *Naegleria* OR *Tetrahymena* OR *Echinamoeba* OR *Paramecium* OR *Balamuthia* OR *Oxytricha* OR *Stylonychia* OR *Diphylleia* OR *Stenamoeba* OR *Singhamoeba* OR *Filamoeba* OR Protozoa OR Protozoan OR Amoeba). The above search terms were modified from the review conducted by Boamah et al. [19]. Figure 1 presents the systematic approach to article inclusion or exclusion. Articles were screened by reading the titles and abstracts and initially excluded if they did not refer to a study that detected *L. pneumophila* and a potential protozoan host from a hospital or potable/drinking water source. Articles were then read in full and excluded if they only described laboratory based simulated or pilot-scale experiments on registered bacterial and protozoan strains.

## 5. Conclusions

Protozoans present in potable water play an important role in *L. pneumophila* survival. Further research is needed to better understand *L. pneumophila*-protozoan interactions and the implications for the prevention of Legionnaires’ disease. To achieve long term disinfection of a water system the control protocols need to be effective against potential hosts harboring *L. pneumophila*. Additionally, an understanding of the mechanisms of VBNC state transformation, and the role of protozoans in this, is needed to effectively evaluate the efficacy of disinfection techniques.

## Figures and Tables

**Table 1:** Potential protozoan hosts of *Legionella pneumophila* isolated from and hospital and potable water systems.

Isolation Source (Temperature at Time of Sampling)	Water Treatment Method	*L. pneumophila*		Potential Protozoan Host		Comments	Country of Origin (Sampling Site)	Reference
		Identification Method	Serogroup Sequence-Type	Genus/Species	Identification Method			
**Hospital Settings**								
Hot (45–52 °C) water tanks	-	Culturing, co-culture assay and serological identification	SG1	*Hartmannella cantabrigiensis* *Vermamoeba vermiformis* *Echinamoeba exudans*	Culturing, light and transmission electron microscopy	Nosocomial legionellosis investigation	USA	[31]
Potable water sites (39–40 °C)	-	Culturing and monoclonal antibody based serotyping	SG1	*Acanthamoeba hatchetti* *Hartmannella cantabrigiensis Vermamoeba vermiformis* *Vahlkampfia* *Filamoeba nolandi* *Comandonia operculata* *Paravahlkampfia ustiana*	Culturing and light microscopy	Nosocomial legionellosis investigation Thermal treatment (70 °C) and chlorination (1.5–2.0 mg/L) controlled the bacteria for 6 months but not amoebae. The treatment reduced incidence of legionellosis	South Dakota, USA	[35]
Cooling tower, humidifier, hot water tank and supply	-	Culturing and co-culture assay	-	*Vermamoeba vermiformis* *Naegleria*	Culturing, light and transmission electron microscopy	-	Paris, France	[32]
Hot (39–60 °C) and cold water supply	-	Culturing (ODR: 1 × 10^3^–9.7 × 10^4^ CFU/L), direct fluorescent antibody and monoclonal antibody based serotyping	SG1 SG5	*Hartmannella* (Hartmannellidae/limax amoebae)	Culturing and light microscopy	Post nosocomial outbreak surveillance	Halifax, Nova Scotia, Canada	[36]
Organ transplant unit hot (mean 56.2 °C) and cold water (mean 16.6 °C) supplies	-	Culturing and serological assay	SG1	*Acanthamoeba* *Hartmannella* *Echinamoeba* *Vahlkampfia* *Tetrahymena* *Vannella*	Culturing and light microscopy	Population density of amoebae was greater in hot water supplies than cold water supplies Along amoebae other diverse eukaryotic microbes were detected as well	UK	[37]
Water supplies	Thermal treatment (60 and 70 °C)	Culturing (*Legionella* ODR: 2.89–6.74 × 10^5^ CFU/L), co-culture, latex agglutination, indirect and immunofluorescence assays, and PFGE	SG1 SG2	*Acanthamoeba* *Vahlkampfia* *Mayorella*	Culturing and light microscopy	Thermal treatment (70 °C) only controlled bacterial contamination for 3 months SG1 is more thermotolerant than SG2 at 60 °C	Germany	[38]
Water network system (mean 56 °C)	-	Amoebae co-culture assay, PCR and sequencing	-	*Vermamoeba vermiformis*	Culturing, PCR and sequencing	Detection of thermotolerant *Vermamoeba vermiformis*	Lausanne, Switzerland	[39]
Water distribution system (18.9–32.6 °C)	Chlorine dioxide treatment Thermal treatment (<50 °C)	Culturing (ODR*: L. pneumophila* SG1: 1 × 10^2^–3.5 × 10^4^ CFU/L and *L. pneumophila* SG2-14: 1 × 10^2^–4 × 10^4^ CFU/L) and latex agglutination assay	SG1 SG2-14	*Acanthamoeba* *Hartmannella*	Culturing and light microscopy	-	Messina, Italy	[40]
Tap water	Chloramine (1.93 ± 1.04 mg/L) treatment	Culturing (protocol: ISO 11731-2:2004, LOD: 1 CFU/100 mL, ODR: 100–1.4 × 10^5^ ± 1.3 × 10^5^ CFU/L), qPCR (LOD: 5 GU, LOQ: 25 GU, *Legionella* ODR: 100–10^9^ gu/L) and EMA-qPCR	ST269	*Acanthamoeba polyphaga*	Culturing, light microscopy and PCR		Italy	[27]
Cold (14.9 °C) and warm (45.1 °C) potable water	Thermal treatment, chlorination (hypochlorates, chloramine), bacterial filters and chlorine dioxide treatment	Culturing (protocols: ISO 11731:1998 and ISO 11731-2:2004, LOD: 1 CFU/100 mL, ODR: 0–3 × 10^3^ CFU/100 mL) and MALDI-TOF MS	-	*Acanthamoeba* *Vermamoeba vermiformis*	Culturing and light microscopy	-	Bratislava, Slovakia	[41]
Cold water system (20–27.3 °C)	Chlorine contents 0.01–0.32 mg/L	qPCR (protocol: ISO/TS 12869:2012, LOD: 5 GU, LOQ: 25 GU, ODR: 2.7–3.8 × 10^2^ gu/L)	-	*Acanthamoeba* *Vermamoeba vermiformis*	Culturing and light microscopy		Johannesburg, South Africa	[42]
Dental unit waterlines	H_2_O_2_ treatment (occasionally)	Heterotrophic plate counts, culturing (protocol: ISO 11731-2:2004, LOD: 1 CFU/100 mL, ODR: 0–2700 CFU/L) and agglutination test	-	*Vermamoeba vermiformis*	Culturing, light microscopy, PCR and sequencing		Italy	[43]
**Potable Water System**								
Unchlorinated water supplies (9.5–13.5 °C)	-	qPCR	-	*Acanthamoeba* *Acanthamoeba polyphaga* *Vermamoeba vermiformis*	qPCR (LOD: 1 cell/reaction), T-RFLP, cloning and sequencing	Along amoebae other diverse eukaryotic microbes were detected as well	Netherlands	[44]
Ground water supplies (5–39 °C)	Aeration, lime stone, granular activated carbon slow sand and rapid sand filtration, ozonisation and pellet softening	Culturing, biofilm batch test and qPCR	-	*Acanthamoeba* *Vermamoeba vermiformis*	18S rDNA sequencing, PCR, T-RFLP and sequencing	Along amoebae other eukaryotic microbes were detected as well	Netherlands	[45]
Water supplies (mean 30 °C)	Reverse osmosis, distillation (82%), chlorination (<0.005–0.2 mg/L), dolomite, limestone and granular activated carbon filtration, fluoride addition (0.3–0.7 mg/L), UV treatment (7.5–35.99 mJ/cm^2^)	Culturing (LOD: 250 CFU/L, *Legionella* ODR: 2.5 × 10^2^–2.5 × 10^5^ CFU/L) and latex agglutination assay	-	*Acanthamoeba* *Vermamoeba vermiformis* *Echinamoeba exundans* *Echinamoeba thermarum* *Neoparamoeba*	qPCR (LOD: 2 C/L, ODR: *Acanthamoeba* < 2–56 C/L and *V. vermiformis* < 2–1670 C/L)	-	Caribbean islands, Leeward Antilles	[46]
Water distribution systems (mean 37.3 ± 8.4 °C)	Chloramine treatment (Chlorine contents 1.8 mg/L), flocculation, sedimentation, and dual-medium filtration	Culturing, qPCR (LOQ: 1–10 copies/reaction, maximum ODR: 13.7 ± 5.1 gc/mL) and T-RFLP	-	*Acanthamoeba* *Vermamoeba vermiformis*	qPCR (LOQ: 1–10 copies/reaction, maximum ODR: *Acanthamoeba* 6.8 ± 2.9 gc/mL and *V. vermiformis* 7.1 × 10^4^ ± 4.4 × 10^3^ gc/mL)	High concentration of chloramine is unable to disinfect water	Southwest Virginia, USA	[47]
Water treatment plant (7–21 °C)	-	Multiplex PCR	-	*Vermamoeba vermiformis*	Culturing, light microscopy, PCR and sequencing	Amoebae were frequently detected at 17 °C	Aragon, Spain	[33]
Water treatment facility (25 ± 3.4–28.2 ± 1.1 °C)	-	PCR (*Legionella* ODR: 1.2 × 10^4^–2.4 × 10^5^ gc/L) and sequencing	-	*Acanthamoeba* *Vermamoeba vermiformis* *Naegleria*	Culturing, PCR, qPCR (ODR: *Acanthamoeba* 2.1 × 10^2^–7.7 × 10^2^ gc/L and *Naegleria* 7.6 × 10^2^–9.4 × 10^2^ gc/L) and sequencing	-	Kaoping River, Taiwan	[48]
Sediments of municipal water storage tank (2.2–28.9 °C)	Chlorination (<4 mg/L)	qPCR (LOD: 2 CE/reaction, *Legionella* ODR: 51 ± 114–7.98 × 10^4^ ± 2.49 × 10^4^ CE/g), cloning and sequencing	SG1	*Acanthamoeba* *Vermamoeba vermiformis*	qPCR (LOD: 2 CE/reaction, ODR: *Acanthamoeba* 22 ± 50–391 ± 243 CE/g and *V. vermiformis* 17 ± 23 CE/g), cloning and sequencing	-	Northeast, East Coast, Midwest, South and West Coast, USA	[49]
Water distribution system	-	qPCR (LOD: 2 CE/reaction, *Legionella* ODR: 2 ± 4–391 ± 17 CE/L), cloning and sequencing	-	*Acanthamoeba* *Acanthamoeba castellanii Vermamoeba vermiformis*	qPCR (LOD: 2 CE/reaction, ODR: *Acanthamoeba* 1 ± 2–16 ± 2 * CE/L and *V. vermiformis* 1 ± 1–9 ± 11 * CE/L), cloning and sequencing	-	USA	[50]
Domestic water systems (mean 20.6 ± 3.8 °C)	-	Culturing, co-culture assay, PCR and sequencing	-	*Vermamoeba vermiformis*	Culturing, light microscopy, PCR and sequencing	-	Geneva, Lausanne and Sion, Switzerland	[51]
Sediments of water storage tank	-	qPCR (ODR: 25 ± 51–300 ± 38 gn/g) and NGS	-	*Acanthamoeba* *Vermamoeba vermiformis*	qPCR (ODR: *Acanthamoeba* 3–7 gn/g, *V. vermiformis* 99 ± 43–120 ± 60 gn/g) and NGS	-	Ohio, West Virginia and Texas, USA	[52]
Potable water	Polyaluminium chloride coagulation, sedimentation, sand and biologically activated carbon filtration and chlorination	qPCR (LOQ: 1–10 copy/reaction, minimum ODR: 3.5 log(gc)/mL)	-	*Acanthamoeba* *Vermamoeba vermiformis*	qPCR (LOD: 1–10 copy/reaction, minimum ODR: 2 log(gc)/mL for *V. vermiformis* and 4 log(gc)/mL for *Acanthamoeba*) and sequencing	Antibiotics (sulfadiazine and ciprofloxacin) promote growth of both bacterium and amoebae	Northern China	[53]
Potable water	Polyaluminium chloride coagulation, sedimentation, sand and biologically activated carbon filtration, chlorination and ozonisation	qPCR (LOQ: 1–10 copies/reaction, minimum ODR ≈ 1 log(gc)/g)	-	*Acanthamoeba* *Naegleria*	qPCR (LOQ: 1–10 copies/reaction, minimum ODR: ≈ 0.5 log(gc)/g for *Naegleria* and ≈ 1 log(gc)/g for *Acanthamoeba*)	Combined chlorination and ozonisation are effective than chlorination only	Northern China	[54]
Potable water	Coagulation, ozonisation, pellet softening, granular activated carbon filtration, rapid and slow sand filtration	Heterotrophic plate counts, culturing (protocol: NEN 6275, LOD: 1 log(CFU)/cm^2^) epifluorescence microscopy, bioluminescence assay, PCR and sequencing	-	*Vermamoeba vermiformis*	qPCR (ODR: 0.7–384 CE/cm^2^)	-	Netherlands	[55]
Residential secondary water supply systems (13.9 ± 4.0–17.4 ± 2.9 °C)	Chloramine treatment (Chlorine contents 0.19–0.89 mg/L)	qPCR (LOQ: 10 copies/reaction, maximum ODR: ≈ 10^2^ gc/mL) and sequencing	-	*Acanthamoeba* *Vermamoeba vermiformis*	qPCR (LOQ: 10 copies/reaction, ODR: 10^1^–10^3^ gc/mL for both *Acanthamoeba* and *V. vermiformis*) and sequencing	-	Shanghai, China	[56]
Water treatment facility	Coagulation, sedimentation, chlorination, ozonisation, granular activated carbon and sand filtration	qPCR (LOQ: 10 copies/reaction, minimum ODR: 10^2^ log(gc)/mL) and sequencing	-	*Vermamoeba vermiformis*	qPCR (LOQ: 10 copies/reaction) and sequencing	Sand filtration after granular activated carbon treatment improves water quality	Southeast China	[57]
Water from private wells after flood	-	Culturing (protocol: ISO 11731, LOD: 1 CFU/100 mL) and qPCR (LOQ: 9.5 gc/mL, maximum ODR: 52.4 gc/mL)	-	*Naegleria fowleri*	qPCR (ODR: 11–610 gc/mL)	-	Louisiana, USA	[58]
Potable water	-	Culturing and DVC-FISH	-	*Acanthamoeba* *Vermamoeba vermiformis*	Culturing and PCR	-	Valencia, Spain	[34]

*Vermamoeba vermiformis* was previously known as *Hartmannella vermiform*. *Paravahlkampfia ustiana* was previously known as *Vahlkampfia ustiana*. ODR: Observed detection range, the amount of bacteria/amoebae/DNA experimentally determined from the samples; CFU/L: colony forming unit/liter; PFGE: pulsed-field gel electrophoresis; PCR: polymerase chain reaction; ISO: International organization for standardization; MALDI-TOF MS: matrix assisted laser desorption ionization-time of flight mass spectrometry; qPCR: quantitative PCR; gu/L: genome unit/liter; LOQ: limit of quantification; LOD: limit of detection; EMA-qPCR: ethidium monoazide-qPCR; T-RFLP: terminal-restriction fragment length polymorphism; C/L: cells/liter; gc/mL: gene copy/milliliter; gc/L: gene copy/liter; CE/reaction: cell equivalent/reaction; CE/g: cell equivalent/gram; CE/L: cell equivalent/liter; * CE/L: cyst equivalent/liter; gn/g: genome copy number/gram; gc/g: gene copy/gram; NGS: next generation sequencing; NEN: Nederlands normalisatie instituut; CE/cm^2^: cell equivalent/cm^2^; DVC-FISH: direct viable count combined with fluorescence in situ hybridization.

**Table 2:** Taxonomic description of potential protozoan hosts.

Hospital Settings	Potable Water System
**Phylum: Amoebozoa** **Class: Tubulinea** Genera: *Vermamoeba*, *Echinamoeba*, *Hartmannella*, *Filamoeba* **Class: Discosea** Genera: *Acanthamoeba*, *Comandonia*, *Mayorella*, *Vannella* **Class: Heterolobosea** Genera: *Vahlkampfia*, *Paravahlkampfia* **Phylum: Percolozoa** **Class: Heterolobosea** Genus: *Naegleria* **Phylum: Ciliophora** **Class: Oligohymenophorea** Genus: *Tetrahymena*	**Phylum: Amoebozoa** **Class: Tubulinea** Genera: *Vermamoeba*, *Echinamoeba* **Class: Discosea** Genera: *Acanthamoeba*, *Neoparamoeba* **Phylum: Percolozoa** **Class: Heterolobosea** Genus: *Naegleria*

**Table 3:** Efficacy of available potable water disinfection protocols on *Legionella pneumophila* and host protozoans.

Organisms	Disinfectant Dose					
	Temperature (°C)	Chlorine (mg-min/L)	Monochloramine (mg-min/L)	Chlorine Dioxide (mg-min/L)	Ozone (mg-min/L)	UV Rays (mJ/cm^2^)
***Legionella pneumophila* studies**						
*Legionella pneumophila* ^1^	70 °C [35,38]	6 mg/L/6 h (5 log reduction) [66]	17 (3 log reduction) [69]	0.4 (3 log reduction) [69]	1–2 mg/L/5 h (5 log reduction) [66]	30 (5 log reduction) ^2^ [66]
***Legionella pneumophila*–potential host protozoans coculture studies**						
*Legionella pneumophila Acanthamoeba* coculture	93 °C ^3^ [63]	>50 mg/L [67]	23 (3 log reduction) [69]	2.8 (3 log reduction) [69]	-	10.8 (4 log reduction) [73]
*Legionella pneumophila* *Vermamoeba* coculture	58 °C [64]	-	-	-	-	-
**Potential host protozoans studies**						
*Acanthamoeba* (trophozoite)	65 °C/10 min (inactivation) [90]	28 (2 log reduction) [79]	40 (2 log reduction) [69]	>5 (2 log reduction) [79]	10 (3 log reduction) [82]	72.2 (3 log reduction) [73]
*Acanthamoeba* (cyst)	80 °C/10 min [81]	3500 (4 log reduction) [82]	352 (2 log reduction) [79]	80 (4 log reduction) [82]	15 (4 log reduction) [82]	800 [91]
*Vermamoeba* (trophozoite)	60 °C/5 min (4 log reduction) [113]	2–4 mg/L/30 min inactivation) [78]	-	-	-	26 (3 log reduction) [73]
*Vermamoeba* (cyst)	60 °C/5 min (2 log reduction) [113]	15 mg/L/10 min (inactivation) [83]	-	-	-	76.2 (3 log reduction) [73]
*Hartmannella* (trophozoite)	53 °C [114]	15 (2 log reduction) [79]	12 2 log reduction) [79]	5 (2 log reduction) [79]	-	-
*Hartmannella* (cyst)	-	156 (2 log reduction) [79]	34 (2 log reduction) [79]	1 (2 log reduction) [79]	-	-
*Naegleria* (trophozoite)	55 °C/15 min [89]	5 (2 log reduction) [79]	4–17 (2 log reduction) [88]	1 (2 log reduction) [79]	6.75 mg/L 30 min (3 log reduction) [84]	24 (4 log reduction) [92]
*Naegleria* (cyst)	65 °C/3 min [89]	29 (2 log reduction) [79]	13 (2 log reduction) [79]	5.5 (2 log reduction) [115]	-	121 (4 log reduction) [92]
*Vahlkampfia* (trophozoite)	-	1 mg/L (inactivation) [77]	-	-	-	-
*Vahlkampfia* (cyst)	-	2 mg/L/2 h (3 log reduction) [77]	-	-	-	-

^1^ Most of the studies focus on culturable bacteria, and non-culturable cells are not estimated. ^2^ No further bacterial inactivation possible, 1–2 × 10^2^ CFU/mL *L.* *pneumophila* remain stable. ^3^ Experiments conducted on *Legionella* sp.
